# Skin metastases from lung cancer: a case report

**DOI:** 10.1186/s13104-015-1105-0

**Published:** 2015-04-11

**Authors:** Laura Pajaziti, Syzana Rexhepi Hapçiu, Shkendije Dobruna, Naim Hoxha, Fisnik Kurshumliu, Artina Pajaziti

**Affiliations:** Department of Dermatology, University Clinical Centre of Kosovo, Prishtina, Republic of Kosovo; Department of Radiology, University Clinical Centre of Kosovo, Prishtina, Republic of Kosovo; Institute of Pathology, University Clinical Centre of Kosovo, Prishtina, Republic of Kosovo; Faculty of Medicine, University Clinical Centre of Kosovo, Prishtina, Republic of Kosovo

**Keywords:** Skin metastases, Lung cancer, Small-cell carcinoma

## Abstract

**Background:**

Lung cancer is one of the most frequent malignancies, with high mortality rates. It can metastasize in almost all organs, but more often invades hilar nodes, liver, adrenal glands, bones and brain. There are various data on the incidence of lung cancer metastases in the skin. In 1-12% of patients with lung cancer are developed skin metastases. Metastases in the skin may be the first sign of lung cancer.

**Case presentation:**

Forty-five years old Albanian male, smoker, was admitted to our department with multiple nodules localized in the skin of the head, neck, back and chest. The nodules measuring 5–15 millimeters in greatest dimension were round and skin-colored, with telangiectasias, firm and tender. They appeared in an eruptive form about two weeks before being admitted at our hospital. In addition, the patient exhibited signs of weight loss, anorexia and fatigue.

Excisional biopsy was performed to one of the lesions. Histopathology confirmed metastatic nature of the lesion namely, malignant tumor of neuroendocrine phenotype consistent with small-cell carcinoma.

Chest X-ray and computed tomography revealed an expansive process in the 7^th^ segment of the left lung, left hilar and mediastinal lymphadenopathy and a suspicious initial secondary deposit in the left adrenal gland. The patient was referred to the department of oncology for further treatment. After the third cycle of chemotherapy, the magnetic resonance imaging revealed brain metastases. The patient passed away four months after the diagnosis of lung cancer first presented with skin metastases.

**Conclusions:**

Metastases in skin may be the first sign of lung cancer. Although rare appearing, we should raise suspicion in cases of atypical lesions in the skin not only of the smokers, but also of the non-smokers. Skin metastases from small-cell lung carcinoma are a poor prognostic indicator. The appearance of multiple skin metastases with other internal metastases shorten the survival time.

## Background

Lung cancer is one of the most frequent malignancies with high mortality rate [1]. It may metastasize in almost all organs, but more frequent sites are hilar nodes, liver, adrenal glands, bones and brain [2,3]. There are various data on the incidence of lung cancer metastases in the skin. According to some data, in men, lung cancer is ranked first with skin metastases (24%), compared to other malignancies [4]. In women, lung cancer is ranked fourth (4%) with skin metastases, following breast cancer, colorectal cancer, melanoma and ovarian cancer [4]. The incidence of skin metastases from lung cancer varies between 1-12% of cases [5]. All histological types of lung cancer may develop metastases in the skin. Metastases from lung cancer may be the first sign of lung cancer and clinically cannot be distinguished from skin metastases originated from other organs [6]. Most common sites of skin metastases from lung cancer are the chest, abdomen, head and neck [6-8]. Clinically, skin metastases occur as round or oval nodules, mobile or fixed, firm, skin-colored (sometimes red, dark red or black). The nodules are usually painless, sometimes may ulcerate. They may rarely appear in the form of solitary or grouped papules, plaque-like, zosteriform, erysipelas-like or as cicatricial alopecia on the scalp [6,9-11]. Compared to other internal malignancies, lung cancer is the fastest in developing skin metastases after initial diagnosis [12]. The occurrence of cutaneous metastases from lung cancer is a poor prognostic indicator [13]. Coexistence of skin metastases with other extracutaneous metastases decreases the average survival time to approximately three months [14]. The survival time can last up to ten months in cases with skin metastases only [15].

## Case presentation

**Figure 1:**
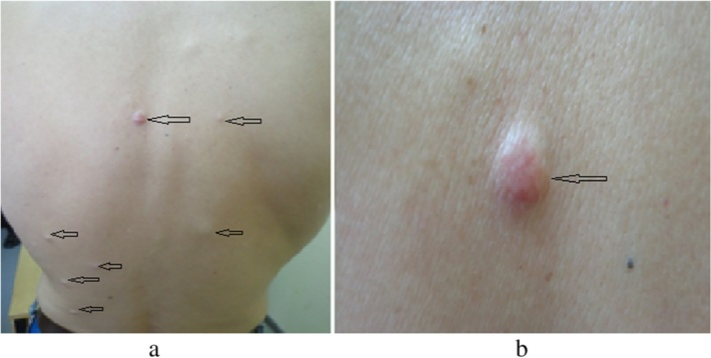
(**a**, **b**) Multiple nodules on the back and a nodule with telangiectasias.

**Figure 2:**
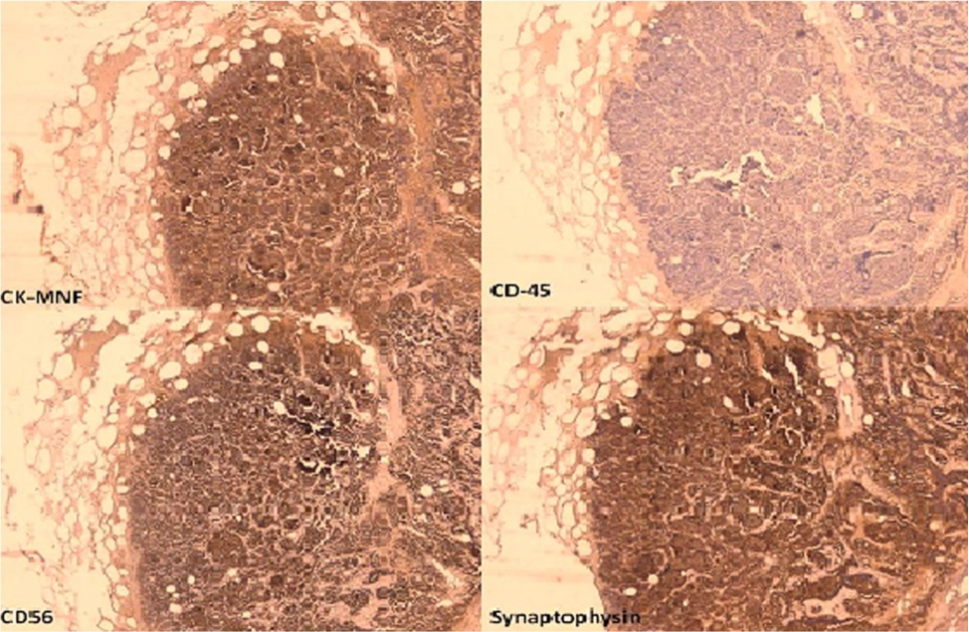
Immunohistochemical stain highlighting neuroendocrine nature of the tumor cells. Cytokeratin 20 (not shown) was negative.

Forty-five years old Albanian male, smoker, was admitted to our department with multiple skin nodules localized in the head, neck, back and chest. The nodules were firm, tender, skin-colored and measured 5-15 millimeters in greatest dimension (Figure 1). They occurred two weeks before being admitted. In addition, the patient exhibited signs of weight loss, anorexia and fatigue, but no symptoms related to respiratory system. Excisional biopsy was performed to one of the lesions. Histopathology confirmed metastatic nature of the lesion namely, malignant tumor of neuroendocrine phenotype consistent with small-cell carcinoma (SCC). The pattern consisted of grouped small cells, oval or fusiform, with large hyperchromatic nuclei. In immunohistochemistry, tumor cells were positive for Pan Cytokeratin (CK-MNF), CD-56 and Synaptophysin. There was no expression of Cytokeratin 7 or Cytokeratin 20. Leukocyte Common Antigen (LCA, CD45) was positive in scattered inflammatory cells of the tumor stroma (Figure 2). Proliferative index as measured by Ki-67 was high (approximately 99% of tumor cells). The bronchoscopic biopsy was nondiagnostic.

**Figure 3:**
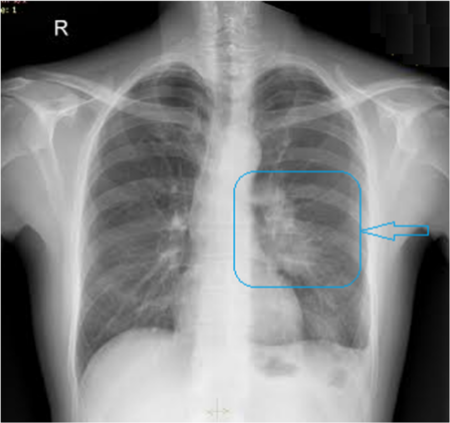
Chest X-ray showed obliteration of the left costophrenic angle and increased inhomogenous density in the left parahilar zone.

Subsequently, chest X-ray (CXR) showed obliteration of the left costophrenic angle and increased inhomogenous density in the left perihilar zone (Figure 3).

**Figure 4:**
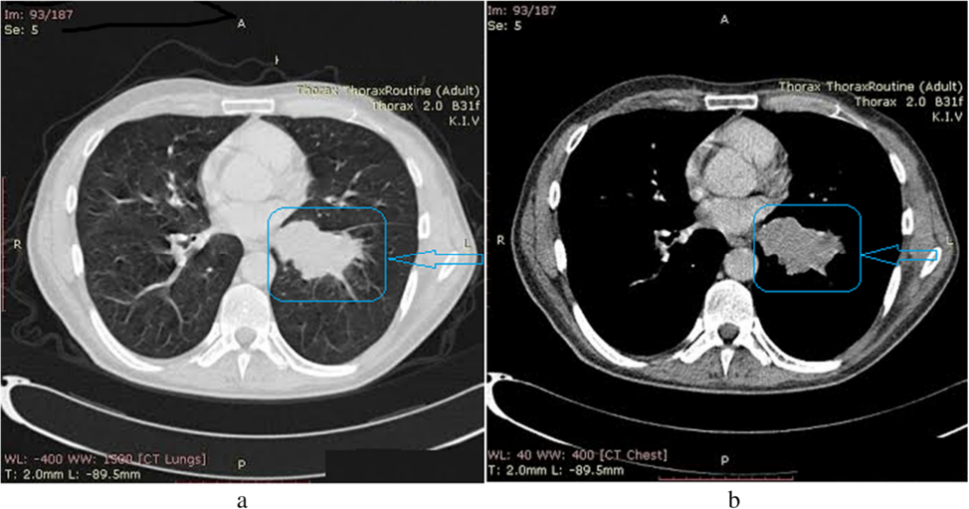
(**a**, **b**) Computed tomography revealed an expansive process in the left lung, left hilar and mediastinal lymphadenopathy.

Computed tomography (CT) revealed an expansive process in the left lung, left hilar and mediastinal lymphadenopathy (Figure 4) and a suspicious secondary initial deposit in the left adrenal gland. Bone scan was unremarkable. Laboratory results were within normal limits, except values of lactate dehydrogenase (LDH) which was slightly elevated namely, 556 U/l (230-460) and C- reactive protein (CRP) with values of 15.4 mg/l (0 to 5.0). The patient was referred to the department of oncology where he underwent chemotherapy with cisplatin 130 mg and etoposide 180 mg with poor clinical response. Meanwhile, skin metastases increased in number and occurred also in the region of the face.

**Figure 5:**
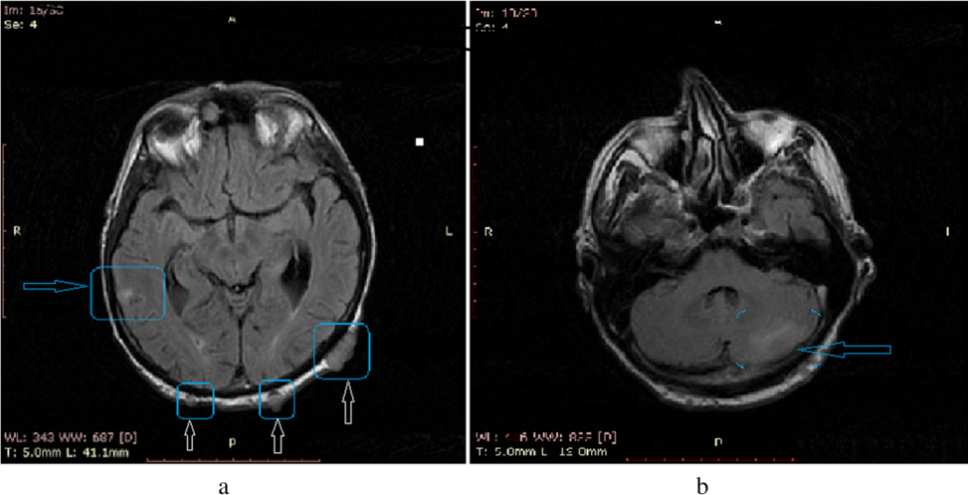
(**a**, **b**) Magnetic resonance imaging of metastases located in the cortex of the temporal lobe, scalp (**a**) and in the left cerebellar hemisphere (**b**).

After the third cycle of chemotherapy, magnetic resonance imaging (MRI) revealed multiple brain metastases located in the cortex of the temporal lobe, left cerebellum, orbital adipose tissue, bilateral extraocular muscles and left lacrimal gland region (Figure 5). The patient passed away four months after the lung cancer diagnosis. Skin metastases were the first sign.

## Discussion

Lung cancer metastases in the skin are rare [3,16]. In literature there are various data regarding the frequency of their development. Patients with lung cancer develop skin metastases in ranges form 1-12% [3,5]. Although it was thought that lung cancer has the highest incidence of skin metastasizing in men [4], recent studies have shown that melanoma leads with skin metastases (32.3%) in men. In women, lung cancer develops metastases in the skin in 2% of cases [17]. It is reported that in 25% of cases of lung cancer, skin is the first site of metastasis [15]. However, in large series of studies, there are reports that the skin is the 13^th^ common site of lung cancer metastasizing [18], while hilar nodes, liver, adrenal glands, bones and brain are more frequent sites [2,3]. In a retrospective study with 80 cases [19], the adjusted skin metastasis prevalence data of various inner cancers indicated that kidney-, lung- and colorectal cancers have a strong positive preference for skin metastatisation. Most skin metastases occur in regions close to the primary cancer. A number of cancers demonstrate a colonization preference to the region of origin: lung cancer to the supradiaphragmatic (mostly chest) and colorectal cancers to the infradiaphragmatic (abdominal) skin regions [19,20]. It was also found that cancer of the upper lobes of the lungs have a greater tendency to metastasizing in skin [21,22]. These findings are clinically relevant and useful especially in patients where skin metastasis is the first indication of a malignancy [20]. Metastasis of human cancer is an organ-selective process that is determined by anatomical and biological factors as well as by specific microenvironmental properties [20]. The precise mechanisms determining the tropism of lung cancer for skin remain unsolved. In small-cell lung cancer, metastatic disease often appears at the time of or shortly after the initial diagnosis [23]. Several recent studies indicate that there are unique genes and gene signatures that modulate specific tropisms of a primary tumor [24]. All histological types of lung cancer may develop metastases in the skin [15]. In literature, various data are reported related to the frequency of skin metastases of different histological types of lung cancer. Some studies demonstrate that adenocarcinoma (ACC) is the most common type of lung cancer that develops skin metastases [4,5,25]. Terashima and Coslett reported in their studies that large-cell carcinoma (LCC) has the highest incidence of skin metastases, followed by ACC. SCC rarely develops metastases in the skin [21,26,27].In our case, the primary cancer was a micro-cellular carcinoma located on the lower left lobe. Skin metastases were the first sign. Although the literature describes cases where metastatic nodules are painless, our patient had painful nodules which made uncomfortable lying position. Cutaneous metastases in the facial region occur in less than 0.5% of patients with metastatic cancer [28]. In a report [28], an unusual case of small-cell lung cancer metastasizing on the face at the time of initial diagnosis is described. In our case, facial metastases occurred during chemotherapy. Metastases in skin occur when the disease has progressed and are indicator of poor prognosis and short survival [2,6,13]. There are reports that the survival time after the appearance of skin metastases is about 5-6 months [3]. Patients that present with skin metastases earlier during the disease course, have poorer prognosis compared to those with later developed metastases [29]. The prognosis is poorer in cases where except cutaneous metastases there are extracutaneous metastases also [14]. Non-resectable primary tumor or small-cell carcinoma is another poor prognostic indicator [15]. Our patient had three poor prognostic indicators: primary micro-cellular inoperable tumor, multiple cutaneous metastases and other distant metastases.

## Conclusion

Metastases in skin may be the first sign of lung cancer. Due to the non- specific appearance, they can be misdiagnosed as benign lesions. In our case, supradiaphragmatic location of non-specific skin lesions, weight loss, and positive smoking history indicated suspicion for lung cancer. Even though skin metastases from lung cancer are rare, we should suspect on it in cases of atypical lesions in the skin in smokers as well as in non smokers. Skin metastases from lung cancer are a poor prognostic indicator. The prognosis is poorer when small-cell lung carcinoma is the primary. The appearance of multiple skin metastases with other internal metastases shorten the survival time.

## Consent

Written informed consent was obtained from the patient’s sister for publication of this Case Report and any accompanying images. A copy of the written consent is available for review by the Editor-in-Chief of this journal.

## Abbreviations

ACC: Adenocarcinoma
CK-MNF: Pan Cytokeratin
CRP: C reactive protein
CT: Computed tomography
CXR: Chest X-ray
LCA: CD45, Leukocyte Common Antigen
LCC: Large-cell carcinoma
LDH: Lactate dehydrogenase
MRI: Magnetic resonance imaging
SCC: Small-cell carcinoma

## References

[1] Rolz Cruz G, Kim CC (2008). Tumor invasion of the skin. Dermatol Clin.

[2] Goljan EF (2006). Rapid Review Pathology.

[3] Mollet TW, Garcia CA, Koester G (2009). Skin metastases from lung cancer. Dermatol Online J.

[4] Brownstein MH, Helwig EB (1972). Metastatic tumors of skin. Cancer.

[5] Hidaka T, Ishii Y, Kitamura S (1996). Clinical features of skin metastases from lung cancer. Intern Med.

[6] Dreizen S, Dhingra H, Chiuten D, Umsawasdi T, Valdivieso M (1986). Cutaneous and subcutaneous metastases of lung cancer. Postgrad Med.

[7] D’Aniello C, Brandi C, Grimaldi L (2001). Cutaneous metastasis from small cell lung carcinoma. Case report. Scand J Plast Reconstr Surg Hand Surg.

[8] Neel V, Sober A, Kufe D, Bast R, Frei E, Holland J, Gansler T, Pollock R, Weichselbaum R (2003). etastatic tumors to the skin. Cancer Medicine.

[9] Ahmed I, Bolognia J, Jorizzo J, Horn T, Mancini A, Mascaro J, Rapini R, Salasche S, Saurat J, Stingl G (2003). Cutaneous Metastases. Dermatology.

[10] Lymphangitic cutaneous metastases from lung cancer mimicking cellulitis (1986). Carcinoma Erysipeloides. West J Med.

[11] Kikuchi Y, Matsuyama A, Nomura K (2001). Zosteriform metastatic skin cancer: report of three cases and review of the literature. Dermatology.

[12] Marcoval J, Moreno A, Peyrí J (2007). Cutaneous infiltration by cancer. J Am Acad Dermatol.

[13] Estarriol MH, Goday MR (2006). Cutaneous Metastases of Small-Cell Lung Cancer. N Engl J Med.

[14] Schoenlaub P, Sarraux A, Groshans E, Heid E, Cribier B (2001). Survival after cutaneous metastasis: a study of 200 cases. Ann Dermatol Venereol.

[15] Ambrogi V, Nofroni I, Tonini G, Mineo TC (2001). Skin metastasis in lung cancer: analysis of a 10-year experience. Oncol Rep.

[16] Ardavanis A, Orphanos G, Ionannidis G, Rigatos G (2006). Skin metastases from primary lung cancer. Report of three cases and a brief review. In vivo.

[17] Lookingbill DP, Spangler N, Helm KF (1993). Cutaneous metastases in patients with metastatic carcinoma: a retrospective study of 4020 patients. J Am Acad Dermatol.

[18] Ask Upmark E (1956). Clinical aspects of tumor metastases. Nord Med.

[19] Kovacs KA, Kenessey I, Timar J. Skin metastasis of internal cancers: a single institution experience. Pathology oncology research: POR. 19(3):515-20. [Pubmed] 10.1007/s12253-013-9611-7 23468362

[20] Kovacs KA, Hedegus B, Kenessey I, Timar J (2013). Tumor type-specific and skin region-selective metastasis of human cancers: another example of the “seed and soil” hypothesis. Cancer Metastasis Rev.

[21] Coslett LM, Katlic MR (1990). Lung cancer with skin metastasis. Chest.

[22] Molina Garrido MJ, Guillén Ponce C, Soto Martínez JL, Martínez Y, Sevila C, Carrato Mena A (2006). Cutaneous metastases of lung cancer. Clin Transl Oncol.

[23] Wan L, Pantel K, Kang Y (2013). Tumor metastasis: moving new biological insights into the clinic. Nat Med.

[24] Kall SL, Koblinski JE (2000). Genes That Mediate Metastasis Organotropism. Madame Curie Bioscience database [Internet].

[25] Perng DW, Chen CH, Lee YC, Perng RP (1996). Cutaneous metastases of lung cancer: an ominous prognostic factor. Zhonghua Yi Xue Za Zhi (Taipei).

[26] Terashima T, Kanazawa M (1994). Lung cancer with skin metastasis. Chest.

[27] De Argila D, Bureo JC, Marquez FL, Pimentel JJ (1999). Small cell carcinoma of the lung presenting as a cutaneous metastasis of the lip mimicking a Merkel cell carcinoma. Clin Exp Dermatol.

[28] Barbetakis N, Samanidis G, Paliouras D, Samanidou E, Tzimorota Z, Asteriou C (2009). Facial skin metastasis due to small-cell lung cancer: a case report. J Med Case Rep.

[29] Ussavarungsi K, Kim M, Tijani L (2013). Skin Metastasis in a patient with Small-Cell Lung Cancer. SWRCCC.

